# PRINS lncRNA Is a New Biomarker Candidate for HPV Infection and Prognosis of Head and Neck Squamous Cell Carcinomas

**DOI:** 10.3390/diagnostics10100762

**Published:** 2020-09-28

**Authors:** Magda Kopczyńska, Tomasz Kolenda, Kacper Guglas, Joanna Sobocińska, Anna Teresiak, Renata Bliźniak, Andrzej Mackiewicz, Jacek Mackiewicz, Katarzyna Lamperska

**Affiliations:** 1 Laboratory of Cancer Genetics, Greater Poland Cancer Centre, 15 Garbary Street, Room 5025, 61-866 Poznan, Poland; mg.kopczynska@gmail.com (M.K.); kacper.guglas@gmail.com (K.G.); a.s.sobocinska@gmail.com (J.S.); anna.teresiak@wco.pl (A.T.); renata.blizniak@wco.pl (R.B.); kasialam@o2.pl (K.L.); 2 Department of Cancer Immunology, Chair of Medical Biotechnology, Poznan University of Medical Sciences, 8 Rokietnicka Street, 60-806 Poznan, Poland; mackiewicz.aa@gmail.com; 3 Postgraduate School of Molecular Medicine, Medical University of Warsaw, 61 Zwirki i Wigury Street, 02-091 Warsaw, Poland; 4 Department of Diagnostics and Cancer Immunology, Greater Poland Cancer Centre, 15 Garbary Street, 61-866 Poznan, Poland; jmackiewicz@ump.edu.pl; 5 Department of Medical and Experimental Oncology, Heliodor Swiecicki Clinical Hospital, Poznan University of Medical Sciences, 16/18 Grunwaldzka Street, 60-786 Poznan, Poland; 6 Department of Oncology, Poznan University of Medical Sciences, Poznan University of Medical Sciences, 82-84 Szamarzewskiego Street, 60-569 Poznan, Poland

**Keywords:** HNSCC, HPV, biomarker, personalized medicine, immune response, microenvironment, viral infection

## Abstract

Numerous studies have shown that human papillomavirus (HPV) infection is one of the important risk factors for head and neck squamous cell carcinoma (HNSCC) progression and affects the expression of multiple genes, which might serve as new biomarkers. This study examines the effects of HPV infection on long non-coding RNA (lncRNA) expression and the immune system, particularly PRINS (Psoriasis susceptibility-related RNA Gene Induced by Stress). The Cancer Genome Atlas (TCGA) expression data for lncRNA genes and clinical data were analyzed by GraphPad Prism 5/7. The expressions of PRINS, CDKN2B-AS1, TTTY14, TTTY15, MEG3, and H19 were significantly different in HPV-positive and HPV-negative patients. HPV-positive patients with high PRINS expression demonstrated significantly better overall survival (OS) and disease-free survival (DFS). HPV-positive patients with high PRINS expression showed changes in gene expression associated with immune and antiviral responses. A majority of HPV-positive patients with high PRINS expression demonstrated a high number of immune cells within tumors. PRINS expression was significantly associated with HPV-infection HNSCC tumors. Validation of these results using data set from Gene Expression Omnibus (GEO) indicated that PRINS is upregulated in HPV active infections and in “atypical 1 (IR)” HNSCC clusters, negatively influencing patients’ overall survival. Patients with high PRINS expression display different immunological profiles than those with low expression levels. For instance, they have active HPV infection status or are clustered in the “atypical 1 (IR)” subtype of HNSCC which influences both viral infection and patients’ survival. It is likely that PRINS could be used as a potential biomarker for HNSCC patients, but its role is dual. On the one hand, it stimulates patients’ immune response, while on the other it can be favorable in virus replication.

## 1. Introduction

Head and neck squamous cell carcinoma (HNSCC) is the sixth leading cancer by incidence worldwide [1]. It is estimated that approximately 600,000 new cases will be diagnosed this year, and about 50% will survive for only five years [2]. The best known risk factors for HNSCC are tobacco and alcohol exposure or dependence. However, over the past decade, there has been an increase in oropharyngeal squamous cell carcinomas (OPCs) associated with the high-risk oncogenic human papillomavirus (HPV) [3]. Patients with HPV-positive (HPV+) OPC are usually middle-aged, non-smoking white men with a history of multiple sexual partners [4].

It is essential to emphasize that HPV(+) and HPV(−) cancers represent different molecular and clinic-pathological characteristics. The prognosis for HPV(+) patients is significantly better than for those with tobacco-related HPV(−) carcinomas in cases of similar treatment [5]. Recently, human papillomavirus has been identified as an independent risk factor for the development of HNSCC [6].

The HPV virus has double-stranded circular DNA that encodes several proteins which affect the host’s cellular program [7]. The infection causes changes in the DNA-repair pathways, mitogenic signaling pathways, cell cycle control, and tumor microenvironment. Molecular heterogeneity within HPV(+) tumors predicts better treatment response [8]. Biological and treatment responses differ between HPV(+) and HPV(−) patients, which might be associated with heterogeneous epigenetic changes in DNA methylation and the functions of non-coding RNAs, such as miRNAs or lncRNAs [9].

Virus-associated epigenetic alterations are likely to be associated with the modulation of long non-coding RNAs (lncRNAs) [10]. lncRNA molecules are defined as non-protein-coding RNAs longer than 200 nucleotides [11]. They can play a variety of regulatory roles, which depend on their cellular localization. In the nucleus, they are involved in chromatin and RNA transformation, while in the cytoplasm they are associated with maintaining mRNA stability, regulating translation, or affecting signaling cascades [12,13,14,15]. lncRNAs can serve as signals of particular cellular states. Thus, they could assist in identifying various pathologies such as cancer. Additionally, they could also potentially provide information on prognosis or responsiveness to chemo- and radiotherapy [12,16,17,18].

HPV is an important factor that affects miRNA modulation and oncogenesis [19,20,21]. However, there is still a lack of comprehensive knowledge of how HPV infection regulates lncRNA expression or the general effect of these phenomena in tumor progression. A few lncRNAs that are closely related to HPV infection and the oncogenesis of HNSCC have been described and could have potential as biomarkers [22,23,24,25,26].

This study focuses on lncRNAs associated with HPV infection: CDKN2B-AS1 (ANRIL), TTTY14 (NCRNA00137), TTTY15 (NCRNA00138), PRINS (Psoriasis susceptibility-related RNA Gene Induced by Stress) (NCRNA00074), XIST (NCRNA00001), MEG3 (NCRNA00023), H19 (NCRNA00008), MALAT1 (NCRNA00047), and CYTOR (NCRNA00152) [10]. Their expression and function were analyzed based on The Cancer Genome Atlas (TCGA) data to understand their role in HNSCC progression.

## 2. Material and Methods

### 2.1. TCGA Analysis

This study applied available cBioPortal tools (http://www.cbioportal.org), gene expression profiles, and clinical data of 522 HNSCC samples from the TCGA project (http://www.cbioportal.org/study?id=hnsc_tcga#summary) [27] and UALCAN resources (http://ualcan.path.uab.edu/index.html) [28]. Due to missing clinical data in cBioPortal, only 81 HPV(−) and 40 HPV(+) HNSCC samples were included in the analysis (Table S1). The HPV (+) samples used for the purpose of this study were selected based on p16 protein expression as principal marker, or in situ hybridization (ISH) marker when the patients were missing p16 information. Study is based on analysis of free available data set and does not need any ethics committee’s agreement and does not violate the rights of other persons or institutions.

The selected samples were divided into two subgroups using the median expression as a cutoff: low lncRNA and high lncRNA, overall survival (OS), as well as disease-free survival (DFS) were analyzed in both groups. The expression level of lncRNAs was correlated with the following clinicopathological parameters: age (<55 vs. >55), gender (female vs. male), smoking category (1+3 vs. 2+4), alcohol consumption (yes vs. no), grade (G1+G2 vs. G3+G4), disease stage (I+II vs. III+IV), T stage (T1+T2 vs. T3+T4), N stage (N0 vs. N1+N2 N3), angiolymphatic invasion (negative vs. positive), and perineural invasion (positive vs. negative).

### 2.2. Genes Associated with Antiviral and Inflammatory Responses

The expression profiles of genes associated with antiviral and inflammatory responses were downloaded from cBioPortal. Next, levels were analyzed in the high and low lncRNA expression groups. UALCAN resources were used to identify genes that are positively and negatively correlated with selected lncRNAs (Pearson correlation >+0.3 or <−0.3, respectively) in both expression groups. The correlated genes were then analyzed using the PANTHER Classification System and placed into specific biological processes and cellular pathways [29]. To determine the differences in expression levels of antiviral and inflammatory response genes, patients were divided into high and low PRINS expression groups using the mean of expression as a cut off. Afterwards, statistical analyses were conducted as described in Section 2.5.

### 2.3. Analysis of the Immune and ESTIMATE Scores

The immune scores and ESTIMATE (Estimation of STromal and Immune cells in MAlignant Tumor tissues using Expression data) scores were downloaded from https://bioinformatics.mdanderson.org/estimate/disease.html. These scores were used to define the infiltration of immune cells into tumor tissues and to infer tumor purity. Statistical analyses were then carried out.

### 2.4. Validation of the Results from TCGA

To validate the obtained results from the TCGA database, the Gene Expression Omnibus (GEO) data repository, with GSE112026 [30] and GSE65858 [31] sets for HNSCC samples, was used. The PRINS expression was compared between normal vs. HPV+ using GSE112026 for 25 and 47 samples, respectively. Using GSE65858 set differences, the next step consisted of comparing differences in the PRINS expression levels between: HPV- and HPV+ (n = 176 vs. 94), HPV- vs. HPV16 vs. other types of HPV (n = 196 vs. 61 vs. 13), as well as between HPV- vs. HPV+(DNA+/RNA+) vs. HPV+(DNA+/RNA+) (n = 196 vs. 35 vs. 19). ROC analysis was applied and later compared between these groups, using GSE112026 data set expression of PRINS based on available clinicopathological parameters such as: age (<60 vs. >60), gender (female vs. male), smoking category (yes vs. no), alcohol consumption (yes vs. no), disease stage (I-III vs. IV), T stage (T1+T2 vs. T3+T4), N stage (N0 vs. N1+N2+N3) cancer category (atypical vs. basal vs. classical vs. mesenchymal). Finally, patients were divided into two groups using the median of PRINS expression as the cut off and OS was calculated. Using this dataset, the validation of expression profiles of antiviral and immune response genes was conducted in two groups: HPV+(DNA+/RNA+) and HPV+(DNA+/RNA-). The analysis was performed as described in Section 2.2 and Section 2.5.

### 2.5. Statistical Analyses

All statistical analyses were performed using GraphPad Prism 5/7 (GraphPad, San Diego, CA, USA). The Shapiro-Wilk normality test, t-test, Mann–Whitney U test or one-way ANOVA test with post-test were used for lncRNA and gene expression according to specified subgroups. All TCGA and GEO data are presented as the mean with standard error of the mean (SEM) or as the median for graphs from UALCAN with lower and upper quartiles. DFS and OS were analyzed using the Log-Rank (Mantel-Cox) and Gehan-Breslow-Wilcoxon tests. The hazard ratio (HR; Mantel-Haenszel) and 95% confidence interval (CI) of the ratio were calculated. In all analyses, *p* < 0.05 was considered as statistically significant.

## 3. Results

### 3.1. Patient Characteristics

In total, 81 HPV(−) and 40 HPV(+) HNSCC samples from TCGA (cBioPortal) with clinical data were included in the analysis. The characteristics of the study groups are presented in Table S1. The expression levels of CDKN2B-AS1, TTTY14, TTTY15, PRINS, XIST, MEG3, H19, MALAT1, and CYTOR were analyzed in normal tissue (taken from healthy tissue near the tumor) and primary tumors resected from HNSCC patients. These lncRNAs had been previously selected for their association with HPV infection [10]. TCGA data (UALCAN database) indicated the up-regulation of CDKN2B-AS1, MALAT1, and CYTOR. However, there was down-regulation of TTTY14 and H19 in cancer tissue in comparison to normal tissue. There were no significant differences in the expression of TTTY15, PRINS, XIST, or MEG3 (Figure S1, Table S2).

### 3.2. Some lncRNAs Are over- or under-Expressed in HPV(+) HNSCC Patients, but Only Patients with High PRINS Expression Have Longer OS

**Figure 1:**
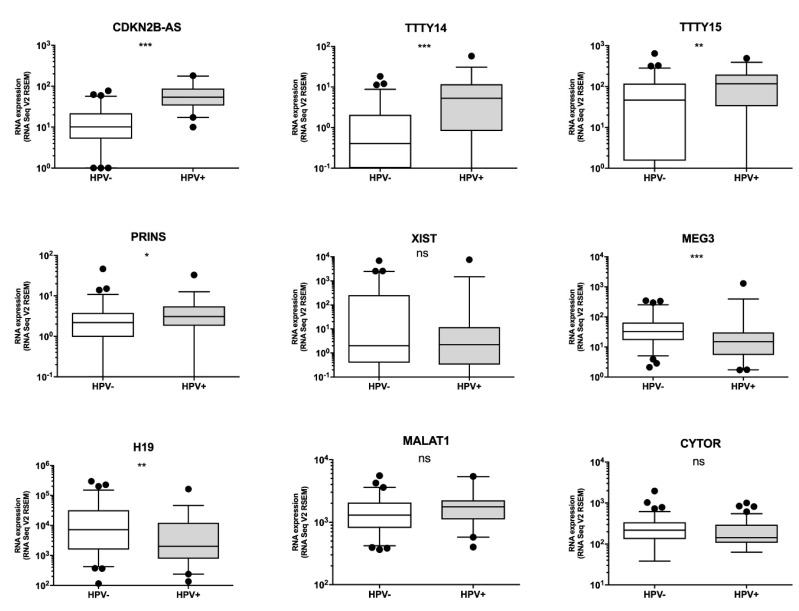
Expression levels of CDKN2B-AS1, TTTY14, TTTY15, PRINS, XIST, MEG3, H19, MALAT1, and CYTOR long non-coding RNAs (lncRNAs) in human papillomavirus (HPV)(−) and HPV(+) head and neck squamous cell carcinoma (HNSCC) patients; un-paired T-test. The graphs show median values with standard error of the mean (SEM). ns: not significant, * *p* < 0.05, ** *p* < 0.01, *** *p* < 0.001.

The analysis of selected lncRNA expression levels in all patients revealed that four of the lncRNAs are up-regulated in HPV(+) patients: CDKN2B-AS1, TTTY14, TTTY15, and PRINS. However, two lncRNAs were down-regulated in HPV(+) patients in comparison to HPV(−) patients: MEG3 and H19. No significant differences were observed in XIST, MALAT1, or CYTOR as shown in Figure 1 and Table S3.

**Figure 2:**
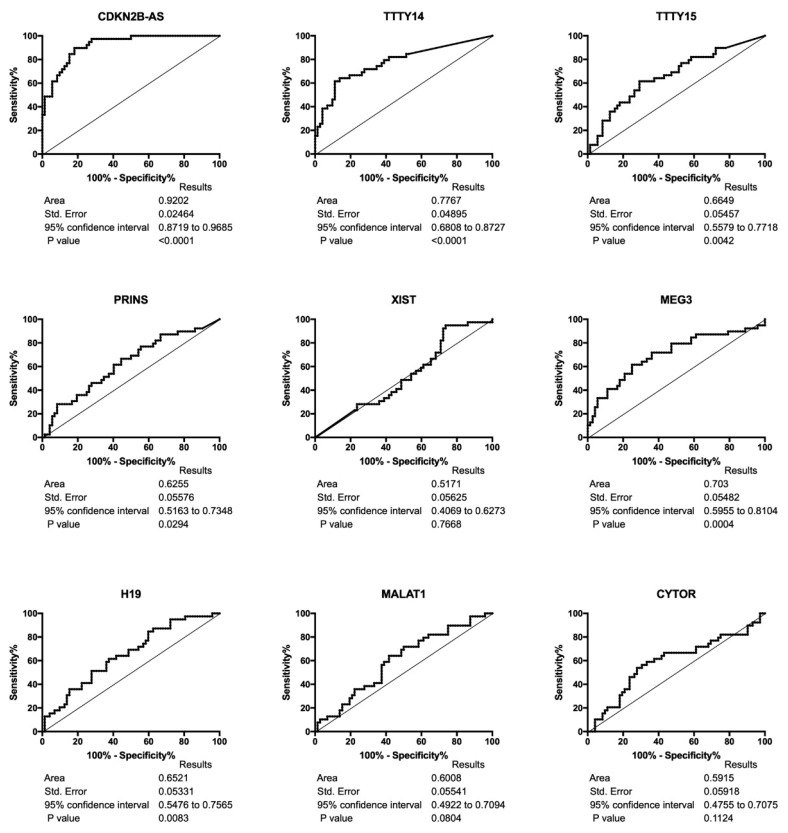
Receiver operating characteristic curve (ROC) analyses of CDKN2B-AS1, TTTY14, TTTY15, PRINS, XIST, MEG3, H19, MALAT1, and CYTOR lncRNAs in HPV(−) and HPV(+) HNSCC patients.

Receiver operating characteristic (ROC) curve analyses showed the high discrimination potential of the following to identify HPV(−) and HPV(+) HNSCC patients: CDKN2B-AS, TTY14, TTY15, PRINS, MEG3, and H19. There were no differences in XIST, MALAT1, and CYTOR, as shown in Figure 2.

**Figure 3:**
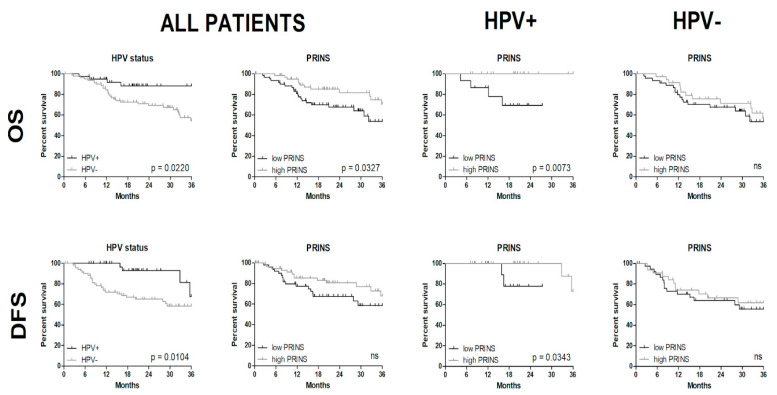
Overall survival (OS) and disease-free survival (DFS) of all patients grouped based on HPV status and PRINS expression as well as between HPV(+) and HPV(−) patients with low and high expression levels of PRINS; log-rank (Mantel-Cox) test; *p* < 0.05 considered as significant.

The OS of the subgroups with low and high expression of CDKN2BAS1, TTTY14, TTTY15, MEG3, and H19 among HPV(−) and HPV(+) patients showed no significant differences (Figure S2). However, a significantly longer OS was found in the HPV(+) group with high PRINS in comparison with the low-PRINS patients (*p* = 0.0073). This relation was observed in the group of high PRINS in comparison to low PRINS patients without taking into consideration HPV status (*p* = 0.0327), as well as in HPV(+) compared to HPV(−) patients according to HPV status only (*p* = 0.0220). Differences in OS were not observed in patients with HPV(−) cancers with high or low PRINS expression (*p* = 0.4947) (Figure 3). Significant differences in DFS were seen between HPV(+) and HPV(−) patients (*p* = 0.0104) and in HPV(+) patients with high versus low PRINS expression levels (*p* = 0.0343), Figure 3.

### 3.3. Clinical Status of HPV(+) HNSCC Patients Is Not Associated with PRINS Expression Level

Analysis of the clinicopathological parameters (age, gender, smoking category, alcohol consumption, grade, T-stage, N-stage, angiolymphatic and perineural invasions) revealed no significant differences (*p* > 0.05) in the expression level of PRINS in the HPV(+) HNSCC patients. However, in all patients, (HPV(−) and HPV(+)), a significant difference in the tumor grade was observed (*p* = 0.0156), as shown in Table S4.

### 3.4. Expression Levels of Viral and Inflammatory Response Genes Are Significantly Different in Patients with High and Low PRINS Expression

**Figure 4:**
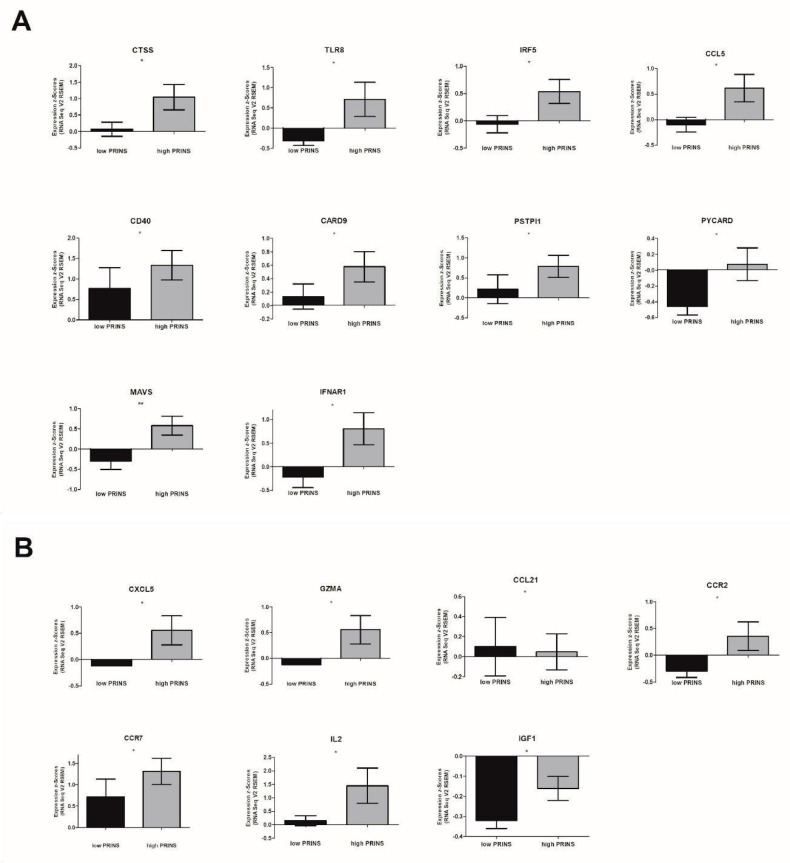
Expression of genes associated with (**A**) antiviral response and (**B**) inflammatory response in HPV(+) HNSCC patients according to PRINS expression level; un-paired T-test or Mann-Whitney U test. The graphs show median values with SEM. Ns: not significant, * *p* < 0.05.

The expression of genes associated with antiviral and inflammatory responses was different between patients with low and high PRINS expression. In patients with high PRINS expression, the up-regulation of the following antiviral response genes was observed: (i) Toll-like receptors and chaperones: CTSS, TLR8; (ii) signaling downstream of Toll-like receptor: IRF5; (iii) Toll-like receptor signaling response genes: CCL5, CD40; (iv) NOD-like receptors and signaling molecules: CARD9, PYCARD, PSTPIP1; (v) signaling downstream of RIG-I-like receptor: MAVS, and (vi) type I interferon signaling: IFNAR1 (Figure 4A and Table S5).

Analysis of the inflammatory response genes revealed significant changes in the following genes: (i) growth factors: IGF1; (ii) immune-stimulatory factors and pro-inflammatory genes: IL2; (iii) chemokines: CXCL5; CCL21; (iv) chemokine receptors: CCR2, CCR7; and enzymatic modulators of inflammation and immunity: GZMA. All of these genes except CCL21 were up-regulated, as shown in Figure 4B and Table S5.

### 3.5. Significant Difference of Immune Cell Infiltration in Tumors of HPV(+) and HPV(−) Patients

**Figure 5:**
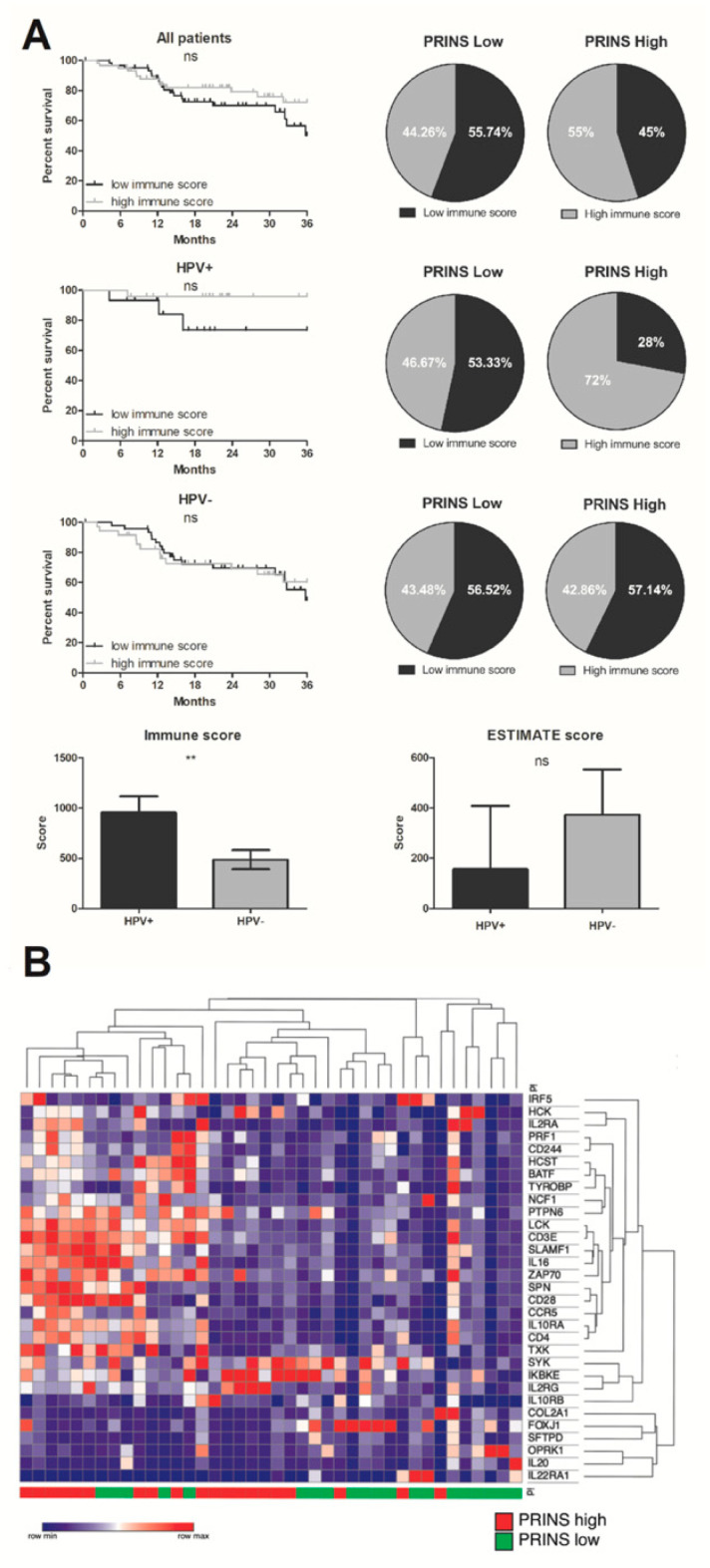
PRINS affects immunophenotype of HNSCC patients; (**A**) OS in all patients (HPV(+) and HPV(−)), HPV(+) and HPV(−) with high and low immune scores based on ESTIMATE tool (Estimation of Stromal and Immune cells in MALignant Tumor tissues), and the percentage of patients with high and low immune scores within the low and high PRINS expression groups; (**B**) heat map and clustering of immune regulatory related genes significantly changed (*p* < 0.05) in low and high PRINS groups of HPV(+) HNSCC patients. The graphs show median values with SEM. Ns: not significant, ** *p* < 0.01.

Immune scores indicated significantly higher infiltration of tumor tissues by immune cells in HPV(+) samples than HPV(−) samples (954.6 ± 162.4 vs. 487.1 ± 96.46, *p* = 0.0099). There were no OS differences associated with immune score in HPV(−) and all patients (*p* = 0.8583, *p* = 0.1742, respectively), but in the HPV(+) group there was slightly better OS for patients with higher immune scores (*p* = 0.0841). Moreover, most HPV(+) patients with high PRINS expression (72%) had higher immune scores than the other groups (Figure 5). The analysis of the ESTIMATE score in HPV(+) and HPV(−) patients demonstrated no significant differences in tumor sample purity.

### 3.6. Validation of PRINS as a Potential Biomarker Using GEO Data

**Figure 6:**
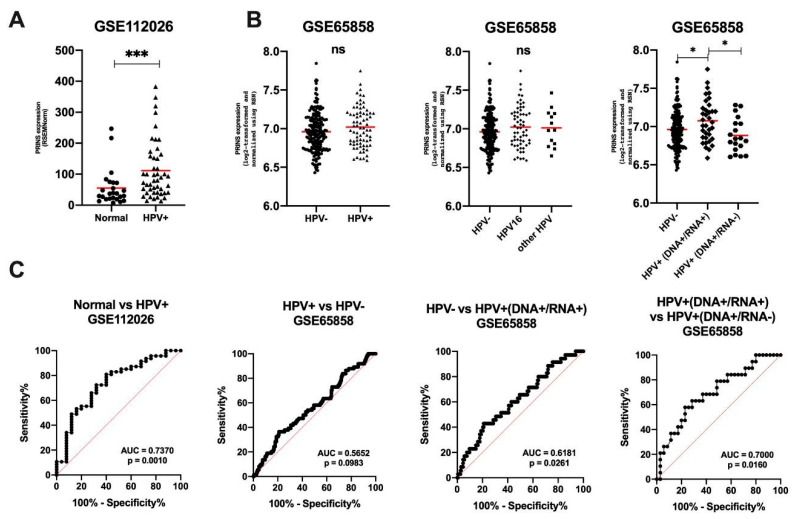
Expression of PRINS in HNSCC patients using GSE112026 and GSE65858 data sets: (**A**) in normal and HPV+ samples; (**B**) depending on HPV status and (**C**) ROC analyses of PRINS’ ability to distinguish groups: normal vs. HPV+, HPV+ vs. HPV−, HPV− vs. HPV+(DNA+/RNA+) and HPV+(DNA+/RNA+) vs. HPV+(DNA+/RNA-); un-paired T-test or one-way ANOVA test with post-test. The graphs show median values; ns: not significant, * *p* < 0.05, *** *p* < 0.001.

To validate results from TCGA, the GEO data for HNSCC patients was used. The significant upregulation of PRINS in HPV(+) HNSCC samples compared to normal levels (*p* = 0.0008) was indicated (Figure 6A). No differences between HPV(−) and HPV(+) (*p* = 0.0985) as well as between HPV(−) vs. HPV16 vs. other types of HPV (*p* = 0.2549) were found. However, expression levels of PRINS were upregulated in the group of HPV+(DNA+/RNA+) vs. HPV(−) (*p* = 0.0401) and HPV+(DNA+/RNA+) vs. HPV+(DNA+/RNA-) (*p* = 0.0247) (Figure 6B). The ROC analysis and estimation of AUC revealed that PRINS with high level of both specificity and sensitivity can be used to distinguish normal and HPV(+) (AUC = 0.7370; 95% CI = 0.6142 to 0.8598; *p* = 0.001). For analysis of HPV(−) vs. all HPV(+), no significant differences were observed (AUC = 0.5652; 95% CI = 0.4884 to 0.6421; *p* = 0.0983). However, when the HPV(+) group was divided due to the presence of virus RNA, the specificity and sensitivity, as well as AUC, were improved: HPV+(DNA+/RNA+) vs. HPV(−) (AUC = 0.6181; 95% CI = 0.5181 to 0.7181; *p* = 0.0261) and HPV+(DNA+/RNA+) vs. HPV+(DNA+/RNA+) (AUC = 0.700; 95% CI = 0.5542 to 0.8458; *p* = 0.0160) (Figure 6C).

Next, based on the GSE65858 data set, an analysis of the clinicopathological parameters was conducted and revealed no significant differences (*p* > 0.05) in the expression level of PRINS in specific clinicopathological parameters groups in the HPV(−), HPV(+), HPV+(DNA+/RNA+) nor HPV+(DNA+/RNA-) of HNSCC patients, apart from the N-stage in the HPV+(DNA+/RNA+) group, where PRINS was upregulated in N1+N2+N3 vs. N0 (7.115 ± 0.050 vs. 6.851 ± 0.076, *p* = 0.044) (Table S6).

All patients (HPV(+) and HPV(−)), HPV(+), HPV+(DNA+/RNA+) and HPV+(DNA+/RNA-) HNSCC patients were divided into two subgroups based on the median of PRINS expression as a cutoff and the patients’ OS between them was assessed. No significant differences between low and high-PRINS expressing groups were observed (Figure S3).

An analysis of antiviral and inflammatory response genes was also conducted. To validate the previous observations, the same set of genes was investigated. In the HPV+ (DNA+/RNA+) group, a significant difference was observed only for CTSS, TLR8 and GZMA genes (Table S6). Surprisingly, the expression levels were higher for groups of patients with low PRINS, which is in direct contrast with former observations in all HPV+ TCGA patients. No differences were observed for the HPV+ (DNA+/RNA-) patients (Table S7) and all HPV+ patients (data not shown).

**Figure 7:**
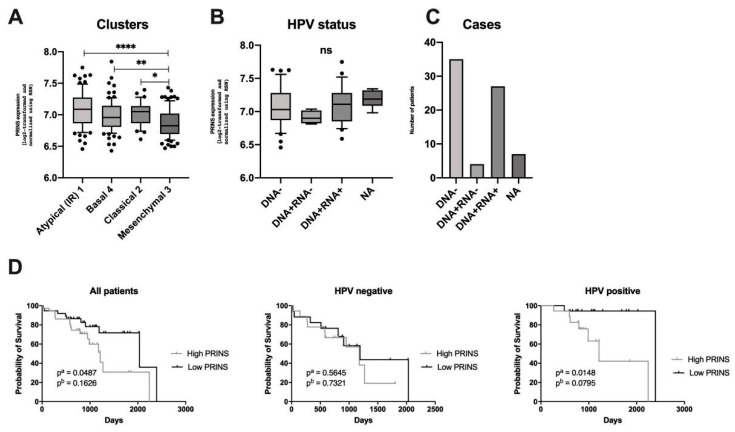
Characteristics of PRINS expression: (**A**) depending on type of cluster and (**B**) viral status in atypical (IR) 1 cluster with (**C**) number of patients’ cases in the atypical (IR) 1 cluster; (**D**) the OS of HNSCC patients depending on the PRINS expression levels in all cases, HPV(−) and HPV(+) from the atypical (IR) 1 cluster. Based on the GSE65858 data set; one-way ANOVA test with post-test; ns: not significant, * *p* < 0.05, ** *p* < 0.01, **** *p* < 0.0001; p^a^—log-rank (Mantel-Cox) test, p^b^—Gehan-Breslow-Wilcoxon Test; *p* < 0.05 considered as significant.

Next, the expression level of PRINS in four different clusters was analyzed. In the case of the “mesenchymal 3” cluster, the lowest expression of PRINS compared to the others was observed, while in “atypical (IR) 1” and “classical 2” the PRINS expression was the highest (*p* < 0.0001) (Figure 7A). In the case of “atypical (IR) 1” no differences in PRINS expression levels regardless of HPV status was indicated (*p* = 0.8010) (Figure 7B). Moreover, in this cluster the number of patients HPV(−) and HPV+ with active infections was similar (Figure 7C). Finally, the OS of patients from “atypical (IR) 1” was checked with regard to PRINS expression levels. In HPV(−) patients, no significant differences in OS were observed. However, in the case of HPV(+) and all analyzed cases, a longer OS in the low-PRINS expressing group was observed (*p* = 0.00148 and *p* = 0.0487, respectively) (Figure 7D).

## 4. Discussion

In the last decade, HPV has been identified as an independent risk factor for HNSCC [6]. Moreover, the number of HPV(+) HNSCC patients is steadily increasing [3,4]. However, in HPV(+) patients, there is still a lack of prognostic biomarkers for specific treatment strategies. In this study, we focused on the selected lncRNAs that were previously identified in HNSCC, cervical cancer patients, or cell lines with HPV infection by gene microarray analysis [32,33,34]. Their biological function and their potential as biomarkers in HNSCC had not been explored previously.

Compared to normal corresponding tissue, TCGA data showed that HNSCC cases had higher expression of CDKN2B-AS1, MALAT1, and CYTOR and lower expression of TTTY14 and H19. Differences in TTTY15, PRINS, XIST, and MEG3 were not found. Analyses of HPV(+) and HPV(−) HNSCC patients indicated that the HPV(+) group had up-regulation of CDKN2B-AS1, TTTY14, TTTY15, and PRINS and down-regulation of MEG3 and H19. Moreover, our ROC analyses showed that CDKN2B-AS, TTTY14, TTTY15, PRINS, MEG3, and H19 could distinguish HPV(+) from HPV(−) samples.

A previous gene expression microarray analysis of HNSCC had pointed out that CDKN2B-AS1, TTTY14, TTTY15, and PRINS are up-regulated in HPV(+) compared to HPV(−) samples, while XIST and CYTOR are down-regulated [32]. In vitro experiments based on HPV16 E6/E7 oncoproteins showed that up-regulation indicated decreased expression of MEG3 and increased expression of H19 in the HFK cell line (human keratinocytes) [33]. Jiang et al. reported that the down-regulation of E6/E7 induces a reduction of MALAT1 in the CaSki cell line (cervical cancer) in vitro [34].

Unfortunately, the available results on lncRNA in HPV(+) HNSCC are limited. Most of the results come from cervical cancer models. Only Nohata et al. showed that 27 and 140 lncRNAs are significantly up-regulated in HPV(+) in comparison with HPV(−) tumors, especially LINC01305, LINC01089 (LIMT), and PTOV1-AS1 lncRNAs [26]. These observations were obtained using an OPC-22 cell-line panel and TCGA data.

Next, we checked the OS of HPV(−), HPV(+), and all patient subgroups with low and high expression of CDKN2BAS1, TTTY14, TTTY15, PRINS, MEG3, and H19. Significantly better OS was observed only in HPV(+) patients with high expression of PRINS. No differences were found between low- and high PRINS-expressing patients with HPV(−) cancers. Lower DFS was parallel to OS in the same HPV(+) patients. This clearly indicates that PRINS could prove to be a prognostic factor for HPV(+) patients. These results and clinicopathological parameters emphasize that PRINS features could be an independent prognostic biomarker for HPV(+) HNSCC patients.

The lncRNA PRINS was first identified by Sonkoly et al. and referred to as Psoriasis susceptibility-related RNA Gene Induced by Stress (PRINS). It is transcribed by RNA polymerase II at different levels in various human tissues, including oral mucosa, and its sequences have homology to stress-induced transcripts, such as heat shock-inducible RNA, G8, and Alu elements. The RNA level of PRINS proved to be increased by stress signals such as UV-B, viral infection (herpes simplex virus), and translational inhibition [35].

The expression of PRINS is also regulated by the proliferation and differentiation states of keratinocytes, and the knock-down of PRINS impairs cell viability after serum starvation in vitro. Moreover, T-lymphokines reduce PRINS expression in the uninvolved psoriatic epidermis, but not in the healthy epidermis. Sonkoly et al. hypothesized that this lncRNA functions as a ribo-regulator and modifies the expression of genes involved in the proliferation and survival of cells exposed to stress [35].

In HeLa cells, the down-regulation of PRINS resulted in changes in cell morphology and altered the gene expression profile (e.g., RAD52, the interferon-inducible, anti-apoptotic G1P3, zinc finger protein 207, annexin A7, and B cell lymphoma 9). Moreover, the up-regulation of PRINS in psoriatic non-lesional epidermis induced the expression of anti-apoptotic G1P3 protein and caused the hyperproliferation of keratinocytes [36]. PRINS is also connected with the development of nephropathy in patients with diabetes [37].

PRINS is also involved in the immune mechanisms, and its expression level is related to chemokines [38]. In a rat model, it affects the immune response and mechanisms of kidney transplant rejection [39]. PRINS has been demonstrated to function as a hypoxia-responsive lncRNA in renal epithelial cells and participates in chemokine CCL5 regulation in hypoxic conditions [38]. Accordingly, we have investigated the expression level of genes associated with antiviral and inflammatory responses in patients with low and high PRINS expression.

We found that PRINS is involved in mounting an antiviral response by affecting Toll-like receptors and chaperones, signaling downstream of Toll-like receptors, signaling response genes, NOD-like receptors, signaling molecules, and signaling downstream of RIG-I-like receptors. It is also connected with type I interferon signaling. Moreover, PRINS modulates inflammation by affecting the expression of growth factors, immune-stimulatory factors, pro-inflammatory genes, chemokine receptors, and enzymatic modulators of inflammation and immunity. Our observations indicate that PRINS is up-regulated during stress and influences local inflammation through the regulation of CCL5, which is well known as a key pro-inflammatory chemokine and is associated with viral infection. It also induces the in vitro migration and recruitment of T cells, dendritic cells, eosinophils, NK cells, mast cells, and basophils, and it supports angiogenesis [40].

Consequently, the ESTIMATE tool was used to elucidate PRINS’ association with the immune state of HNSCC patients. Patients with high immune scores tended to have better survival but for HPV(+) only. Importantly, this observation was not indicated for HPV(+) with low PRINS expression. A previous report based on TCGA and GEO databases analyzed tumor-infiltrating immune cells in HPV(+) and HPV(−) HNSCC patients. The results showed that HPV(+) patients have more infiltrating B and T cells and fewer neutrophils. T cell subtypes revealed that cytotoxic T cell subtypes predominated in HPV(+) HNSCC, and the ratio of M1/M2 macrophages was much higher in this group. Moreover, enhanced infiltration of B cells and CD8+ T cells was identified as an independent protective factor, while high neutrophil infiltration was a risk factor for HPV(+) HNSCC patients [41]. Our analyses suggest that patients with high levels of PRINS have a more enhanced local immune microenvironment, which is manifested by longer OS and the expression of specific markers.

The classification of HPV(+) HNSCC patients into better- and worse-prognosis subgroups is already known. However, there is still a lack of biomarkers to stratify patients for the implementation of more precise treatment. Zhang et al. indicated that HPV(+) patients could be divided into two distinct subtypes: HPV-KRT (with high expression of genes in keratinocyte differentiation and the oxidation-reduction process) and HPV-IMU (with strong immune responses, e.g., high expression of CD40 and mesenchymal differentiation). The HPV-IMU patients exhibit better prognosis, but the OS analysis did not show significant differences between HPV-IMU and HPV-KRT HNSCC patients [42]. Our study confirms the higher expression of immune response genes such as CD40 and CD4 in the better-responding-high PRINS group of HPV-IMU described by Zhang et al.

To validate our results, we used available data from GEO and observed that PRINS is indeed upregulated in both HPV(+) (when compared to normal samples) and in HPV+(DNA+/RNA+), which is assessed as active HPV infection [31] in opposition to HPV(−) and HPV+(DNA+/RNA-), which are described as being inactive infection. Moreover, ROC analysis revealed that PRINS with high sensitivity and specificity can distinguish active and inactive viral infection in HNSCC patients. Wichmann et al., based on their own data and results used also in this paper (GSE65858), postulated that HPV diagnostics based on an estimation of specific viral DNA and RNA is better than checking the expression of p16. They also indicated that molecular profiles of HPV+(DNA+/RNA-) patients are similar to HPV(−) and HPV+(DNA+/RNA+) tumors are molecularly and clinically distinct from HPV(−). Moreover, molecular profiling of patients indicated four different subgroups (“atypical 1 (IR)”, “classical 2”, “basal 4” and “mesenchymal 3”), where the atypical type was characterized by enriched genes connected with cell cycle and immune response [31]. Our analysis, based on the GEO database, indicated that PRINS expression is high in “atypical 1 (IR)”, “classical 2” and “basal 4” subgroups, compared to the mesenchymal subtype of HNSCC. It is difficult to compare this result to the one obtained from the TCGA database, where high PRINS expression was connected with favorable immune phenotype. Unfortunately, the “atypical 1 (IR)” group (like the three others) is not homogeneous in terms of HPV infection and in it HPV(−) as well as HPV(+), both active and inactive cases, can be found. However, it should be emphasized that in the “atypical 1 (IR)” subgroup of patients, the HPV active cases is highest in comparison to the other subgroups and the proportion of the active HPV to negative cases is the same [31]. The genes selected, based on TCGA analysis, were not validated in terms of the PRINS expression in HPV(+) HNSCC patients in the corresponding group (HPV+(DNA+/RNA+)) from the GSE65858 set. Moreover, analysis of OS in correlation with PRINS level also does not support the observation, based on TCGA data. The reason for this result is unknown and cannot readily be explained. However, it is difficult to clearly compare the TCGA and GEO data because, in both pieces of research, different detection methods of HPV infection were used. Some authors have indicated that immunohistochemistry of p16 alone is not the best method for estimation of HPV infection and should be combined with HPV DNA status as well as Ki-67 or pRB expression [43,44,45]. Surprisingly, when we analyzed the OS in the “atypical 1 (IR)” cluster, we observed that patients with low levels of PRINS have longer survival time than patients with a higher expression. It is likely that patients with higher PRINS have higher infection status and more progressed disease caused by viral replication and upregulation of cell cycle genes which generally conclude in worse patients outcome.

The available data and our results are intriguing in terms of the implementation of PRINS lncRNA in clinical practice as a biomarker. PRINS lncRNA is upregulated in HPV(+) patients and connected with active HPV infection. The lack of evidence of the variable PRINS expression among clinical parameters could suggest a connection to better or worse patient survival. We postulate that PRINS could be responsible for the regulation of the immune system through the modulation of inflammation as well as the stimulation of viral replication. However, this hypothesis needs experimental verification based on in vitro and in vivo models.

## 5. Conclusions

In HPV(+) HNSCC patients’ tumors four lncRNAs (CDKN2B-AS1, TTTY14, TTTY15 and PRINS) and two (MEG3 and H19) are up- or down-regulated, respectively as compared with HPV(−) patients. The specified profiling allows for HPV(+) and HPV(−) differentiation. Patients with high PRINS expression display different immunological profiles than those with low expression and have active HPV infection status or are clustered in the “atypical 1 (IR)” subtype of HNSCC.

## Supplementary Materials

The following are available online at https://www.mdpi.com/2075-4418/10/10/762/s1, **Figure S1:** Expression levels of CDKN2B-AS1, TTTY14, TTTY15, PRINS, XIST, MEG3, H19, MALAT1, and CYTOR lncRNAs in normal tissue (*n* = 44) and primary tumor (*n* = 520) of HNSCC patients; graphs from UALCAN database, modified; Un-paired T-test. The graphs show median values presented as transcripts per million with lower and upper quartiles of boxes. ns: not significant, * *p* < 0.05, **** *p* < 0.0001; **Figure S2:** OS in all patients, HPV(+) and HPV(−) low and high expression levels of CDKN2B-AS1, TTTY14, TTTY15, MEG3 and H19 lncRNAs; a - Log-rank (Mantel-Cox) test; *p* <0.05 considered as significant; **Figure S3:** OS of all, HPV (+), HPV+ (DNA+/RNA+) and HPV+ (DNA+/RNA-) patients with low and high expression levels of PRINS based on GSE65858 data set; p^a^—log-rank (Mantel-Cox) test, p^b^—Gehan-Breslow-Wilcoxon Test; *p* <0.05 considered as significant; **Table S1:** Characteristics of TCGA HNSCC patients examined in this study; **Table S2:** The expression levels of lncRNAs in cancer and normal tissues. Data from UALCAN database; **Table S3:** The expression levels of lncRNAs in HPV(+) vs. HPV(−) patients; un-paired T-test or Mann-Whitney U test. *p* < 0.05 considered as significant; **Table S4:** The expression level of PRINS in HPV(−), HPV(+), and all both HPV(−) and HPV(+) HNSCC patients according to clinical-pathological parameters; un-paired T-test; *p* < 0.05 considered as significant; **Table S5:** Expression of genes associated with antiviral response and inflammatory response in HPV(+) HNSCC patients according to PRINS expression level based on TCGA data; un-paired T-test or Mann-Whitney U test. *p* < 0.05 considered as significant; **Table S6:** The expression level of PRINS in the HPV(−), HPV(+), HPV+(DNA+/RNA+) and HPV+(DNA+/RNA-) HNSCC patients according to clinical-pathological parameters based on GSE65858 data set; ND—no data; un-paired T-test or one way ANOVA with post test; *p* < 0.05 considered as significant; **Table S7:** Expression of genes associated with antiviral response and inflammatory response in HPV+ (DNA+/RNA+) and HPV+ (DNA+/RNA-) HNSCC patients according to PRINS expression level; un-paired T-test or Mann-Whitney U test. *p* < 0.05 considered as significant.

## Abbreviations

HNSCC	Head and Neck Squamous Cell Carcinoma
HPV	Human Papillomavirus
lncRNA	long non-coding RNAs
ncRNAs	non-coding RNAs
OS	overall survival
DFS	disease free survival
TCGA	The Cancer Genome Atlas
OPC	oropharyngeal squamous cell carcinoma
miRNAs	microRNAs
PRINS	Psoriasis associated non-protein coding RNA induced by stress
CDKN2B-AS1	cyclin-dependent kinase inhibitor 2B antisense non-coding RNA
TTTY14	testis-specific transcript, Y-linked 14
TTTY15	testis-specific transcript, Y-linked 15
MEG3	maternally expressed 3
H19	H19 imprinted maternally expressed transcript
XIST	X inactive specific transcript
MALAT1	metastasis associated lung adenocarcinoma transcript 1
CYTOR	cytoskeleton regulator RNA
ESTIMATE	Estimation of STromal and Immune cells in MAlignant Tumor tissues
CI	Confidence Interval
CTSS	cathepsin S
TLR8	toll-like receptor 8
IRF5	interferon regulatory factor 5
CCL5	C-C motif chemokine ligand 5
CD40	cluster of differentiation 40
CARD9	caspase recruitment domain family member 9
PYCARD	PYD and CARD domain containing
PSTPIP1	proline-serine-threonine phosphatase interacting protein 1
MAVS	mitochondrial antiviral signaling protein
IFNAR1	interferon alpha and beta receptor subunit 1
IGF1	insulin like growth factor 1
IL2	interleukin 2
CXCL5	C-X-C motif chemokine ligand 5
CCL21	C-C motif chemokine ligand 21
CCR2	C-C motif chemokine receptor 2
CCR7	C-C motif chemokine receptor 7
GZMA	granzyme A
