# Supplementary material for: PRINS lncRNA Is a New Biomarker Candidate for HPV Infection and Prognosis of Head and Neck Squamous Cell Carcinomas
Source: Diagnostics (Basel). 2020 Sep 28;10(10):762. doi: 10.3390/diagnostics10100762 (PMC7599931; doi:10.3390/diagnostics10100762)
Supplement: Supplementary file 1 [file diagnostics-10-00762-s001.pdf]

SUPPLEMENTARY MATERIAL:

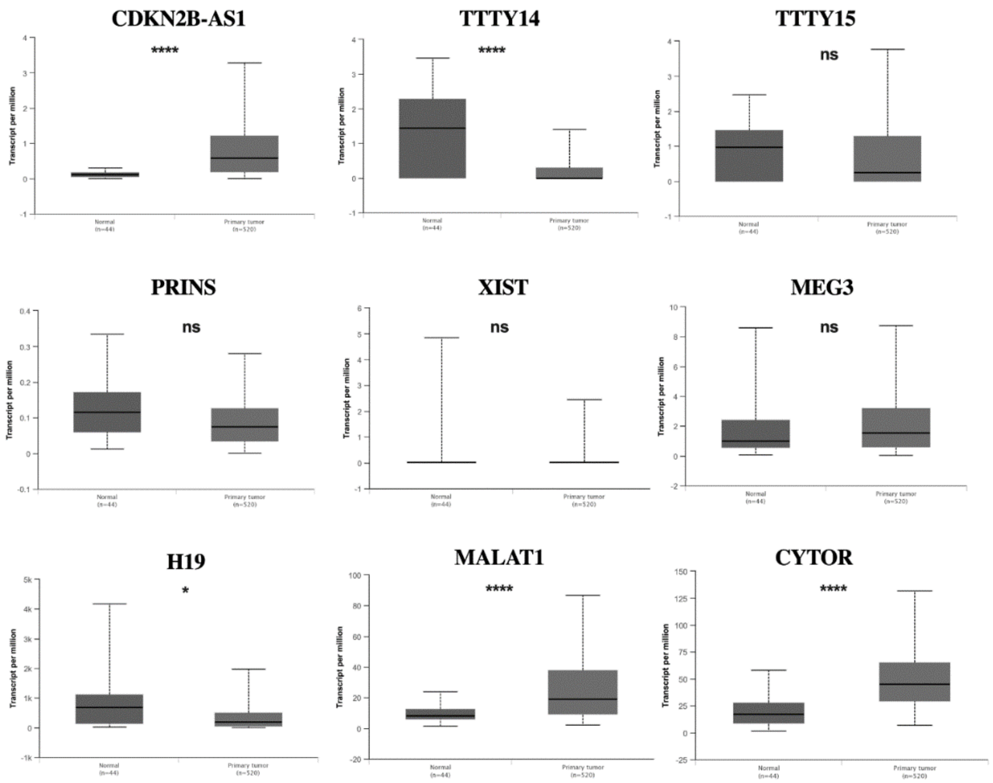

Figure S1. Expression levels of CDKN2B-AS1, TTTY14, TTTY15, PRINS, XIST, MEG3, H19, MALAT1, and CYTOR lncRNAs in normal tissue (n = 44) and primary tumor (n = 520) of HNSCC patients; graphs from UALCAN database, modified; Un-paired T-test. The graphs show median values presented as transcripts per million with lower and upper quartiles of boxes. ns: not significant, * $p < 0.05$, **** $p < 0.0001$.

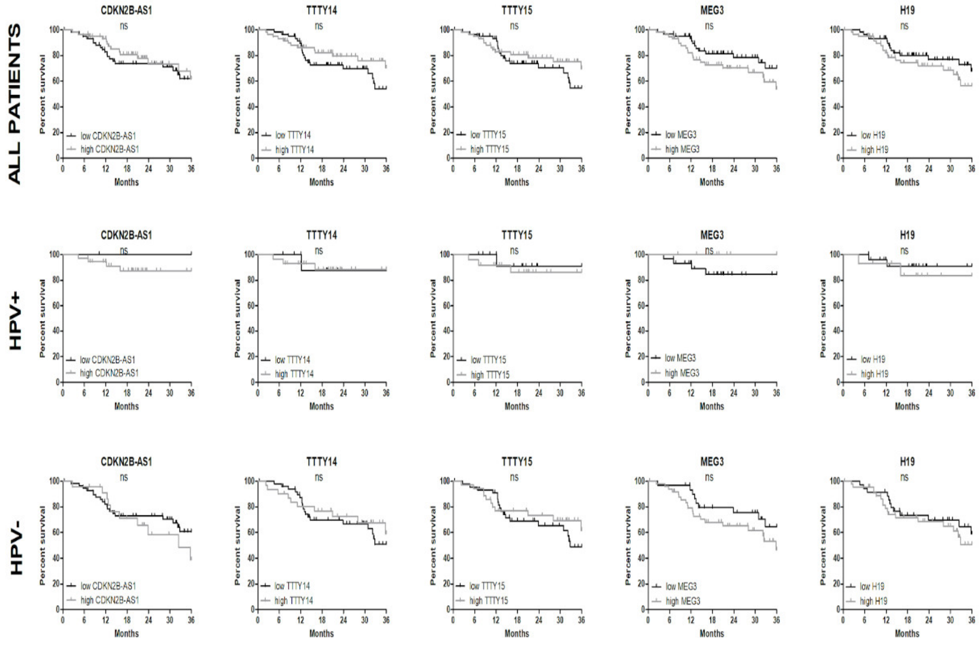

Figure S2. OS in all patients, HPV(+) and HPV(-) low and high expression levels of CDKN2B-AS1, TTTY14, TTTY15, MEG3 and H19 lncRNAs; a - Log-rank (Mantel-Cox) test; $p < 0.05$ considered as significant.

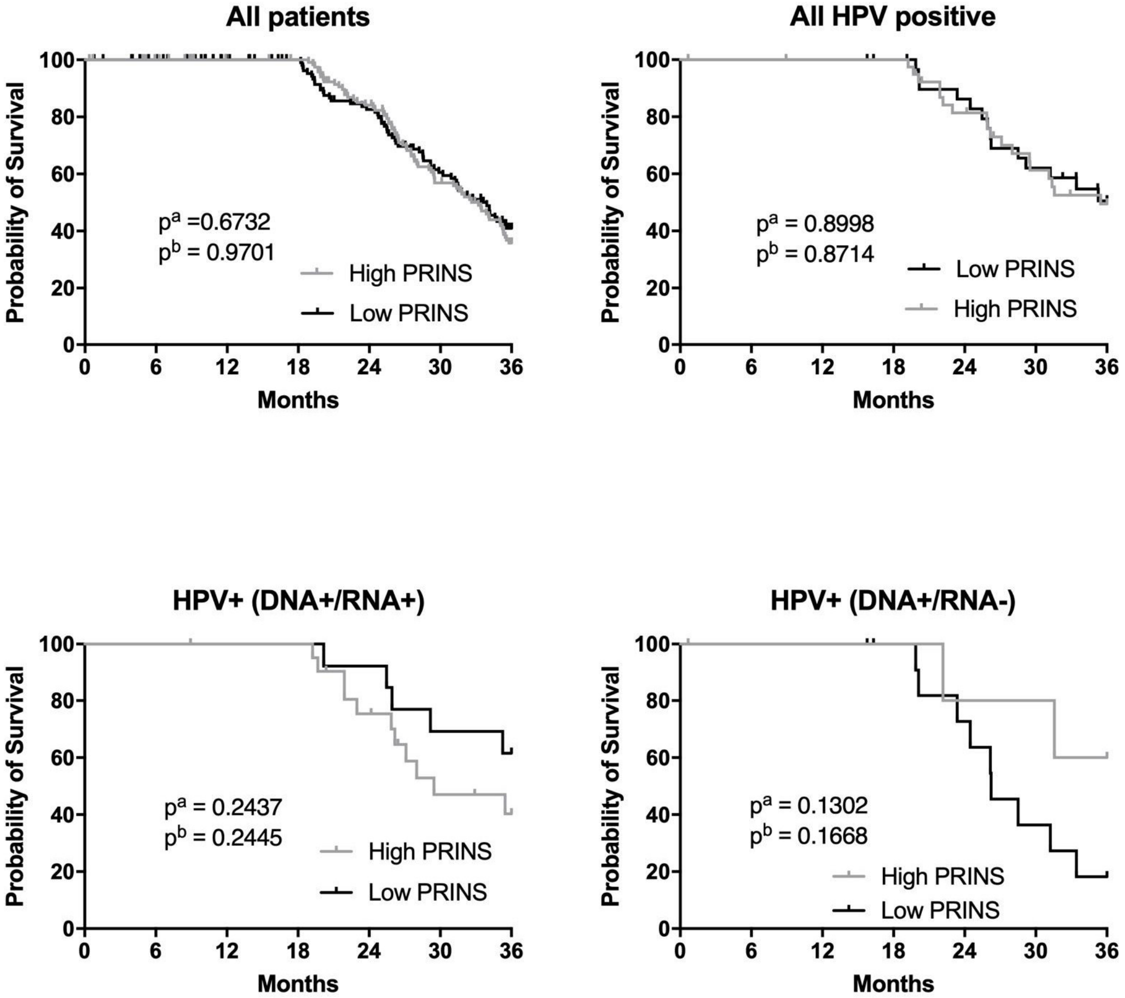

Figure S3. OS of all, HPV (+), HPV+ (DNA+/RNA+) and HPV+ (DNA+/RNA-) patients with low and high expression levels of PRINS based on GSE65858 data set; p^a - log-rank (Mantel-Cox) test, p^b - Gehan-Breslow-Wilcoxon Test; $p < 0.05$ considered as significant.

Table S1. Characteristics of TCGA HNSCC patients examined in this study.

**Parameter**		**HPV(-)** [cases]	**HPV(+)** [cases]	**ALL (HPV(-) and HPV(+))** [cases]
**Age**	<55	25	16	41
	>55	56	24	80
	ND	0	0	0
**Gender**	Female	20	4	24

	Male	61	36	97
	ND	0	0	0
**Smoking**	Yes	58	24	82
	No	23	15	38
	ND	0	1	1
**Alcohol consumption**	Yes	60	30	90
	No	19	9	28
	ND	2	1	3
**Grade**	G1+G2	59	15	74
	G3+G4	20	15	35
	ND	2	10	12
**T-stage**	T1+T2	23	16	39
	T3+T4	50	7	57
	ND	8	17	25
**N-stage**	N0	29	8	37
	N1+N2 +N3	49	31	80
	ND	3	1	4
**Perineural invasion**	Yes	44	3	47
	No	31	14	45
	ND	6	23	29
**Angiolymphatic invasion**	Yes	32	10	42
	No	36	6	42
	ND	13	24	37

ND – no data

Table S2. The expression levels of lncRNAs in cancer and normal tissues. Data from UALCAN database.

	Tumor vs. Normal **[mean]**	p-val
CDKN2B-AS1	0.588 vs. 0.107	1.11e-16
TTTY14	0 vs. 1.445	5.59e-07
TTTY15	0.243 vs. 0.966	2.40e-01
PRINS	0.074 vs. 0.116	4.11e-01
XIST	0.004 vs. 0.002	6.73e-01
MEG3	1.557 vs. 0.996	2.39e-01
H19	185.012 vs. 686.186	3.90e-02
MALAT1	9.077 vs. 8.03	1.64e-12
CYTOR	44.913 vs. 17.175	1.63e-12

Table S3. The expression levels of lncRNAs in HPV(+) vs. HPV(-) patients; un-paired T-test or Mann-Whitney U test. $p < 0.05$ considered as significant.

	HPV(+) vs HPV(-) **[mean ± SEM]**	p-val
CDKN2B-AS1	66.81 ± 6.905 vs. 15.77 ± 1.915	<0.0001
TTTY14	8.861 ± 1.882 vs. 1.652 ± 0.3215	0.0001
TTTY15	134.0 ± 18.47 vs. 79.93 ± 12.35	0.0042
PRINS	4.488 ± 0.8757 vs. 3.324 ± 0.686	0.029
XIST	42.5 ± 197.7 vs. 433.7 ± 123.5	0.7679
MEG3	69.50 ± 34.96 vs. 59.73± 8.734	0.0004
H19	10820 ± 4278 vs. 29251 ± 633	0.0084
MALAT1	1964 ± 195.3 vs. 1585 ± 120.8	0.0810
CYTOR	247.7± 37.49 vs. 284.3 ± 32.04	0.113

Table S4. The expression level of PRINS in HPV(-), HPV(+), and all both HPV(-) and HPV(+) HNSCC patients according to clinical-pathological parameters; un-paired T-test; $p < 0.05$ considered as significant.

**Parameter**	**HPV(-)** **[mean ± SEM]**	**HPV(+)** **[mean ± SEM]**	**ALL (HPV(-) and HPV(+))** **[mean ± SEM]**

**Age**	<55	3.976 ± 1.824	4.904 ± 0.8498	4.338 ± 1.152
	>55	3.228 ± 0.4314	4.421 ± 1.326	3.586 ± 0.4980
	p-val	0.1754	0.1324	0.9367
**Gender**	Female	2.837 ± 0.7743	3.787 ± 2.188	2.995 ± 0.7226
	Male	3.663 ± 0.7998	4.706 ± 0.9280	4.050 ± 0.6087
	p-val	0.4464	0.6521	0.1946
**Smoking**	Yes	4.039 ± 0.8612	3.831 ± 0.5510	3.978 ± 0.6281
	No	1.995 ± 0.3400	5.992 ± 2.106	3.573 ± 0.8984
	p-val	0.0691	0.7728	0.2579
**Alcohol consumption**	Yes	3.569 ± 0.8148	4.337 ± 1.099	3.825 ± 0.6527
	No	3.315 ± 0.7937	4.947 ± 0.9376	3.840 ± 0.6247
	p-val	0.6299	0.0989	0.1990
**Grade**	G1+G2	2.926 ± 0.4346	3.365 ± 0.8026	3.015 ± 0.3809
	G3+G4	5.101 ± 2.208	6.338 ± 2.024	5.631 ± 1.514
	p-val	0.1569	0.1300	**0.0156**
**T-stage**	T1+T2	2.218 ± 0.3501	3.979 ± 0.8318	2.941 ± 0.4167
	T3+T4	4.307 ± 0.9924	2.352 ± 0.9174	4.067 ± 0.8800
	p-val	0.2212	0.1813	0.7882
**N-stage**	N0	3.076 ± 0.6522	3.771 ± 1.042	3.227 ± 0.5543
	N1+N2+N3	3.860 ± 0.9663	4.866 ± 1.076	4.250 ± 0.7216
	p-val	0.5081	0.8076	0.3604

**Perineural invasion**	Yes	3.127 ± 0.4740	1.793 ± 0.9082	3.042 ± 0.4486
	No	4.219 ± 1.504	3.324 ± 0.7869	3.940 ± 1.060
	p-val	0.6130	0.3447	0.8974
**Angiolymphatic invasion**	Yes	2.698 ± 0.3100	2.937 ± 0.5081	2.755 ± 0.2629
	No	4.431 ± 1.364	2.073 ± 1.061	4.094 ± 1.182
	p-val	0.7963	0.1927	0.4027

Table S5. Expression of genes associated with antiviral response and inflammatory response in HPV(+) HNSCC patients according to PRINS expression level based on TCGA data; un-paired T-test or Mann-Whitney U test. $p < 0.05$ considered as significant.

**Gene**	**Low PRINS [mean ± SEM]**	**High PRINS [mean ± SEM]**	**p-val**
**CTSS**	0.06551 ± 0.2194	1.046 ± 0.3853	0.0193
**TLR8**	-0.3091 ± 0.1186	0.7104 ± 0.4217	0.0499
**IRF5**	-0.06304 ± 0.1593	0.5362 ± 0.2212	0.0315
**CCL5**	-0.09860 ± 0.1441	0.6168 ± 0.2683	0.0167
**CD40**	0.7695 ± 0.5067	1.333 ± 0.3574	0.0294
**CARD9**	0.1301 ± 0.1872	0.5753 ± 0.2273	0.0385
**PYCARD**	-0.4627 ± 0.1056	0.07362 ± 0.2079	0.0315
**PSTPIP1**	0.2176 ± 0.3614	0.7888 ± 0.2799	0.0439
**MAVS**	-0.3029 ± 0.1992	0.5812 ± 0.2314	0.0062
**IFNAR1**	-0.2207 ± 0.2215	0.8031 ± 0.3413	0.0133
**IGF1**	-0.3198 ± 0.04003	-0.1609 ± 0.05973	0.0193
**IL2**	0.1516 ± 0.1857	1.449 ± 0.6512	0.0359
**CXCL5**	-0.1227 ± 0.02848	-0.1885 ± 0.007108	0.0468
**CCL21**	0.1003 ± 0.2905	0.04864 ± 0.1800	0.0468
**CCR2**	-0.2963 ± 0.1196	0.3550 ± 0.2659	0.0337
**CCR7**	0.7152 ± 0.4145	1.312 ± 0.3078	0.0411

**GZMA**	-0.1237 ± 0.1679	0.5555 ± 0.2758	0.0468

Table S6. The expression level of PRINS in the HPV(-), HPV(+), HPV+(DNA+/RNA+) and HPV+(DNA+/RNA-) HNSCC patients according to clinical-pathological parameters based on GSE65858 data set; ND - no data; un-paired T-test or one way ANOVA with post test; p < 0.05 considered as significant.

**Parameter**		**HPV(-) [mean ± SEM]**	**HPV(+) [mean ± SEM]**	**HPV+ (DNA+/RNA+) [mean ± SEM]**	**HPV+ (DNA+/RNA-) [mean ± SEM]**
**Age**	<60	6.958 ± 0.025	6.991 ± 0.040	6.993 ± 0.061	6.87 ± 0.079
	>60	6.966 ± 0.027	7.065 ± 0.048	7.137 ± 0.071	6.9 ± 0.067
	p-val	0.834	0.232	0.087	0.782
**Gender**	Female	6.953 ± 0.043	7.038 ± 0.075	7.078 ± 0.122	6.786 ± 0.087
	Male	7.009 ± 0.020	7.018 ± 0.033	7.075 ± 0.048	6.902 ± 0.058
	p-val	0.286	0.787	0.978	0.712
**Smoking**	Yes	6.993 ± 0.020	7.023 ± 0.062	7.076 ± 0.053	6.879 ± 0.063
	No	6.956 ± 0.044	7.023 ± 0.035	7.08 ± 0.095	6.894 ± 0.096
	p-val	0.493	0.995	0.967	0.898
**Alcohol consumption**	Yes	6.993 ± 0.020	7.017 ± 0.033	7.086 ± 0.053	6.883 ± 0.054
	No	6.958 ± 0.060	7.06 ± 0.077	7.041 ± 0.101	ND
	p-val	0.555	0.633	0.705	ND
**UICC stage**	I-III	6.977 ± 0.030	6.953 ± 0.070	6.953 ± 0.109	6.906 ± 0.105
	IV	6.952 ± 0.023	7.044 ± 0.034	7.12 ± 0.048	6.874 ± 0.060
	p-val	0.506	0.206	0.051	0.765
**T-stage**	T1+T2	6.955 ± 0.027	7.029 ± 0.046	7.021 ± 0.071	6.902 ± 0.083
	T3+T4	6.965 ± 0.025	7.017 ± 0.041	7.152 ± 0.049	6.871 ± 0.068
	p-val	0.791	0.848	0.059	0.770
**N-stage**	N0	6.962 ± 0.029	6.936 ± 0.062	6.851 ± 0.076	6.943 ± 0.105
	N1+N2+N3	6.961 ± 0.023	7.042 ± 0.034	7.115 ± 0.050	6.857 ± 0.058
	p-val	0.840	0.186	**0.044**	0.467
**Cancer type**	Atypical	7.077 ± 0.052	7.091 ± 0.042	7.094 ± 0.055	6.913 ± 0.051
	Basal	6.983 ± 0.030	6.995 ± 0.072	6.966 ± 0.157	6.971 ± 0.159
	Classical	7.031 ± 0.036	6.959 ± 0.133	ND	6.877 ± 0.149

Mesenchymal	6.848 ± 0.038	6.913 ± 0.056	7.056 ± 0.102	6.829 ± 0.082
p-val	**<0.0001**	0.100	0.781	0.748

Table S7. Expression of genes associated with antiviral response and inflammatory response in HPV+ (DNA+/RNA+) and HPV+ (DNA+/RNA-) HNSCC patients according to PRINS expression level; un-paired T-test or Mann-Whitney U test. $p < 0.05$ considered as significant.

	**HPV+ (DNA+/RNA+)**				**HPV+ (DNA+/RNA-)**		
**Gene**	**Low PRINS [mean ± SEM]**	**High PRINS [mean ± SEM]**	**p-val**		**Low PRINS [mean ± SEM]**	**High PRINS [mean ± SEM]**	**p-val**
**CTSS**	6.947 ± 0.05971	6.752 ± 0.02964	**0.049555**		6.739 ± 0.07882	6.560 ± 0.07528	0.967068
**TLR8**	6.433 ± 0.03290	6.329 ± 0.01647	**0.003138**		6.392 ± 0.02920	6.310 ± 0.03734	0.126616
**IRF5**	6.956 ± 0.1123	7.019 ± 0.1116	0.608948		6.796 ± 0.08950	6.799 ± 0.08497	0.901427
**CCL5**	10.48 ± 0.1839	10.08 ± 0.2184	0.203840		9.068 ± 0.2406	9.377 ± 0.4534	0.710209
**CD40**	6.867 ± 0.04359	6.759 ± 0.03042	0.102314		6.738 ± 0.05494	6.715 ± 0.04476	0.967068
**CARD9**	7.371 ± 0.1035	7.267 ± 0.09957	0.399998		7.079 ± 0.1345	7.144 ± 0.1476	0.710209
**PYCARD**	7.080 ± 0.05669	6.956 ± 0.05827	0.137459		6.979 ± 0.05523	7.086 ± 0.05994	0.200592
**PSTPIP1**	6.781 ± 0.06053	6.717 ± 0.06409	0.418734		6.648 ± 0.06484	6.644 ± 0.1194	0.710209
**MAVS**	6.751 ± 0.03083	6.808 ± 0.03849	0.346890		6.929 ± 0.05850	6.925 ± 0.08645	0.967068
**IFNAR1**	7.849 ± 0.04888	7.834 ± 0.06572	0.881931		7.658 ± 0.09744	7.791 ± 0.09238	0.302001
**IGF1**	6.600 ± 0.04231	6.509 ± 0.05539	0.116944		6.820 ± 0.1647	6.572 ± 0.09475	0.482764
**CXCL5**	6.720 ± 0.1246	6.660 ± 0.04850	0.437977		7.203 ± 0.2645	6.784 ± 0.2316	0.090508
**CCL21**	7.396 ± 0.2294	7.273 ± 0.2215	0.655911		8.130 ± 0.4038	7.184 ± 0.2243	0.090508
**CCR7**	8.003 ± 0.1600	7.656 ± 0.1765	0.109436		7.693 ± 0.3671	7.196 ± 0.3835	0.482764
**GZMA**	9.036 ± 0.1931	8.586 ± 0.1444	**0.049555**		7.980 ± 0.2833	7.869 ± 0.2736	0.649723
